# Outcome following local injection of a liquid amnion allograft for treatment of equine tendonitis or desmitis – 100 cases

**DOI:** 10.1186/s12917-022-03480-5

**Published:** 2022-11-07

**Authors:** Hugh. R. Duddy, Mike. J. Schoonover, Brent. A. Hague

**Affiliations:** 1 Department of Veterinary Clinical Sciences, College of Veterinary Medicine, Oklahoma State University, Stillwater, OK USA; 2 Oakridge Equine Hospital, Edmond, OK USA

**Keywords:** Regenerative medicine, Horse, Tendon, Ligament

## Abstract

**Background:**

Tendon and ligament injuries are significant causes of loss of use and early retirement in performance horses. Amniotic fluid and tissue are excellent sources of growth factors and cytokines important in tendon and ligament healing. Thus, an equine-origin liquid amnion allograft (ELAA) may be beneficial in the treatment of equine tendonitis and desmitis. Objectives of this study were to report the outcome achieved (i.e. ability to return to work) for horses diagnosed with tendonitis or desmitis lesions treated with local injection of ELAA and to compare these outcomes to those reported for other regenerative medicine modalities.

**Methods:**

A prospective, multi-center, non-blinded clinical trial was conducted. Equine veterinarians at 14 sites were selected to participate in the data collection for the trial. Criterion for inclusion was a horse presenting with lameness which was attributed to tendonitis or desmitis by diagnostic anesthesia and/or imaging. These horses were subsequently treated by local injection of the lesion with ELAA by the attending veterinarian. Standardized questionnaires describing each horse’s signalment, discipline, ability to return to work, and any adverse events were completed and submitted by the attending veterinarian following a minimum of six months of follow-up. The current literature was reviewed to identify clinical studies reporting outcomes of equine tendonitis/desmitis lesions treated with other regenerative therapies. Contingency table analyses were performed comparing outcomes.

**Results:**

Questionnaires for 100 horses with 128 tendonitis and desmitis lesions met the inclusion criteria. Of these, 72 horses with 94 lesions returned to or exceeded their original level of work, 10 horses with 13 lesions returned to work but could not perform to previous standards, and 18 horses with 20 lesions did not return to work as a result of the injury. No differences were observed when outcome of horses treated with ELAA were compared to those of similar studies using other regenerative therapies.

**Conclusions:**

Treatment of tendonitis and desmitis lesions by local injection of ELAA resulted in similar outcomes for horses returning to previous level of performance as other regenerative modalities such as mesenchymal stem cells, platelet-rich plasma, and autologous conditioned serum; however, blinded placebo-controlled studies are indicated.

**Supplementary Information:**

The online version contains supplementary material available at 10.1186/s12917-022-03480-5.

## Background

Tendon and ligament injuries are a significant cause of morbidity in the performance horse and often compromise return to the previous level of activity [1–8]. Healing of these injuries progresses through several sequential, overlapping phases [9, 10]. The initial response is the acute inflammatory phase characterized by pain, intra-lesional hemorrhage, and edema. Circulating inflammatory cells infiltrate the injury and release initial growth factors and cytokines. Growth factors and cytokines orchestrate biological processes throughout the healing process [1, 9]. The acute inflammatory response is followed by the proliferative phase where fibroblastic cells produce scar tissue comprised of far more type III collagen than the original tendon [1, 3, 10, 11]. The remodeling phase follows, resulting in the gradual transformation to more type I collagen though not to the same proportion as the original pre-injury tissue [1, 3, 10]. The end result is a tendon that lacks pre-injury biomechanical properties [1, 3, 9, 10].

Regenerative medicine seeks to restore structure and function to injured tissue [12–14]. The main components of regenerative therapies are scaffold, growth factors, and cells [12–14]. Current therapies include mesenchymal stem cells (MSCs) [7, 10, 12–17], platelet rich plasma (PRP) [12, 14, 15, 18–23], and autologous conditioned serum (ACS) [12, 14, 15, 24, 25]. Local injection of these therapies is common practice for horses with tendinous and ligamentous injuries. Amnion is considered a significant source of growth factors and cytokines important in tissue regeneration and healing [26, 27]. An equine origin, liquid amnion allograft (ELAA; RenoVō™, Equus Innovations) comprised of amnion and amniotic fluid is commercially available. Off-the-shelf availability of a regenerative modality could facilitate clinical use avoiding the time delay currently associated with autologous therapies that require sample collection and processing. To the authors’ knowledge, no published studies evaluate ELAA for the treatment of equine tendonitis or desmitis. The objective of this clinical trial was to evaluate the outcome (i.e. ability of horse to return to work) following local injection of ELAA for the treatment of tendinitis and desmitis lesions. Additionally, these outcomes were to be compared to those reported in published clinical studies using regenerative modalities such as MSCs, PRP, ACS, and gene therapies for the treatment of tendonitis and desmitis. Our hypothesis was that treatment of specific equine tendonitis and desmitis lesions with local injection of ELAA would result in outcomes comparable to horses with similar lesions treated with other regenerative modalities.

## Methods

The design of the study was a non-blinded clinical trial. Experienced equine veterinarians at 14 hospitals (sites) were selected for participation in the study. An aseptically manufactured, commercially packaged, acellular, cryopreserved ELAA derived from equine amniotic tissues of healthy mares (RenoVō™, Equus Innovations) was used by all attending veterinarians. The ELAA was shipped frozen by the manufacturer to the sites with instructions to keep the product frozen at -80˚C until use to preserve the beneficial proteins. Each site was provided with a calibrated -80˚C portable freezer (Shuttle C Model ULT-25NE, Stirling Ultracold) to store the ELAA when received. The ELAA was provided in 1.5 mL and 3.0 mL volumes aseptically packaged in individual vials. Instructions for use directed the veterinarian to thaw the ELAA at room temperature and administer it locally in or around the lesion using strict aseptic technique including aseptic preparation of the administration site and the use of sterile gloves and administration paraphernalia. The volume of ELAA used to treat an individual lesion was left to the attending veterinarian’s discretion.

Client-owned horses were selected for enrollment and subsequent treatment with ELAA at the discretion of the attending veterinarian following examination, diagnosis and client education. Written informed client consent for treatment was obtained by the attending veterinarian prior to treatment. Criteria for inclusion in the study was defined as a horse displaying fore or hind limb lameness attributed to a specific diagnosis (lesion) of tendonitis or desmitis proven or supported by diagnostic anesthesia and/or imaging (ultrasound or magnetic resonance imaging) of the regional anatomy. Additionally, the identified lesion(s) must have been treated by local injection of ELAA with follow-up information available for a period of six months or more. All horses remained in the care of the owners throughout the study period.

For each horse enrolled, a standardized questionnaire was completed by the attending veterinarian which included the date of ELAA administration, ELAA product code and serial number, horse identification and signalment, diagnosis(es), duration of injury/lameness, initial and re-evaluation lameness grade [28], ability to return to work, adverse events, and product satisfaction (Additional file 1). The studied outcome measure was the ability of the horse to return to work following ELAA treatment. This was categorized as either returning to or exceeding the horse’s previous level of work, returning to work but not able to perform to the horse’s previous standards, or inability to return to work as a result of the injury and showing no improvement. Following review of the completed questionnaires, information from those meeting the above criteria was compiled and those deemed not to have met the study criteria or those missing pertinent information (diagnosis, initial lameness grade, or ability to return to work) were excluded. If a horse was administered ELAA for treatment of two or more separate lesions concurrently, each was considered a separate lesion.

### Statistical analysis

The current literature was reviewed to identify clinical studies reporting outcomes of equine tendon and ligament lesions treated with regenerative medicine modalities including MSCs, PRP, ACS, and gene therapies. Contingency tables were used to calculate a Pearson Chi-square value for comparison of outcomes between these prior studies and those of the current study. For any comparison in which one or more cells contained less than 5 observations, a Fisher’s exact test was used. A *p* < 0.05 was considered significant.

## Results

### Outcome data

Nineteen veterinarians submitted 170 questionnaires describing their experiences with ELAA in horses. Following review, 100 questionnaires describing outcomes for 100 horses were determined to be complete and to have met the study’s inclusion criteria; only these were included in the assessment (Table 1). A total of 128 tendonitis or desmitis lesions were described in these 100 questionnaires: 78/100 questionnaires described local injection of a single lesion, 18/100 described local injection of two lesions, 2/100 described local injection of three lesions, and 2/100 questionnaires described local injection of four lesions. Two of the 100 horses were treated twice for the same lesions, one horse with a single lesion and the other with two lesions. Overall, 72/100 horses (72%) were reported to have either returned to or exceeded their previous level of work following local injection with the ELAA: 18/72 horses at less than three months, 40/72 horses at between three and six months, and 14/72 horses at greater than six months. Ten out of the 100 horses (10%) were reported to have returned to work but not perform to previous standards: 2/10 horses at less than three months, 6/10 horses at between three and six months, and 2/10 horses at greater than six months. Eighteen of the 100 horses (18%) were reported to have not returned to work as a result of the injury and showed no improvement.

**Table 1:** Outcome by signalment of 100 horses with tendonitis/desmitis treated with an equine origin, liquid amnion allograft

	Returned to or exceeded previous level of work	Returned to work but could not perform to previous standards	No return to work as a result of the injury and no improvement
Age			
≤ 3 years	4	0	3
4 to 10 years	40	6	9
≥ 11 years	26	4	5
ND	2	0	1
Weight			
< 500 kg	8	3	2
500 – 800 kg	59	7	15
> 800 kg	3	0	0
ND	2	0	1
Breed			
AQHA/APHA	38	2	17
Arabian/½ Arabian	17	3	0
Warmblood	9	1	0
Thoroughbred	2	2	1
Other	4	2	0
ND	2	0	0
Sex			
Stallion	4	0	0
Mare	31	4	6
Gelding	36	6	12
ND	1	0	0
Discipline			
Western Performance			
Barrel racing	14	1	4
Roping	5	1	4
Cutting	4	0	3
Reining	3	0	4
Not specified	2	0	0
Show	22	4	1
Sport			
Racing—TB	2	2	1
Racing—Other	4	0	0
Hunter/jumper	6	2	0
Dressage	2	0	0
Not specified	1	0	0
Other	2	0	1
ND	5	0	0
Location of Injury			
Fore	47	6	13
Hind	25	4	5
Pre-Treatment Lameness Grade			
1	7	0	3
2	27	1	2
3	33	6	11
4	5	3	2
Duration of Injury			
< 6 months	37	4	10
6 Months—1 Year	13	3	3
1—2 years	6	1	3
> 2 years	10	1	0
ND	6	1	2

*ND* Not documented, *TB* Thoroughbred

Duration of injury was reported on the questionnaire as < 6 months for 51/100 horses, 6 months to 1 year for 19/100 horses, 1 to 2 years for 10/100 horses, and > 2 years for 11/100 horses, while the duration of injury was not reported for 9/100 horses (Table 1). The individual lesions were categorized based on their anatomic location and ability of the horse to return to work (Table 2). Horses associated with 94 of the 128 lesions were reported to have either returned to their previous level of work or exceeded their previous level of work: horses associated with 20/94 lesions at less than three months, 53/94 lesions at between three and six months, and 21/94 lesions at greater than six months. Horses associated with 13 of the 128 lesions were reported to have returned to work but not perform to previous standards: horses associated with 2/13 lesions at less than three months, 6/13 lesions at between three and six months, and 5/13 lesions at greater than six months. Horses associated with 21 of the 128 lesions were reported to have not returned to work as a result of the injury and showed no improvement.

**Table 2:** Outcome of 128 tendonitis/desmitis lesions in 100 horses treated with an equine origin, liquid amnion allograft

	Returned to or exceeded previous level of work	Returned to work but could not perform to previous standards	No return to work as a result of the injury and no improvement
Suspensory branch desmitis			
All	25 (66%)	4 (10%)	9 (24%)
Fore	16 (73%)	0 (0%)	6 (27%)
Hind	9 (56%)	4 (25%)	3 (19%)
Proximal suspensory desmitis			
All	11 (73%)	1 (7%)	3 (20%)
Fore	5 (83%)	1 (17%)	0 (0%)
Hind	6 (67%)	0 (0%)	3 (33%)
DDF tendonitis – foot/pastern region			
All	15 (68%)	3 (14%)	4 (18%)
Fore	12 (66%)	3 (17%)	3 (17%)
Hind	3 (75%)	0 (0%)	1 (25%)
DDF tendonitis – cannon region			
All	8 (100%)	0 (0%)	0 (0%)
Fore	5 (100%)	0 (0%)	0 (0%)
Hind	3 (100%)	0 (0%)	0 (0%)
SDF tendonitis			
All	10 (67%)	2 (13%)	3 (20%)
Fore	8 (62%)	2 (15%)	3 (23%)
Hind	2 (100%)	0 (0%)	0 (0%)
SDF-AL desmitis			
Fore	1 (50%)	1 (50%)	0 (0%)
Collateral desmitis – coffin joint			
Fore	9 (90%)	1 (10%)	0 (0%)
Collateral desmitis—Other			
All	3 (60%)	1 (20%)	1 (20%)
Fore	2 (67%)	0 (0%)	1 (33%)
Hind	1 (50%)	1 (50%)	0 (0%)
Straight sesamoidean desmitis			
All	6 (86%)	0 (0%)	1 (14%)
Fore	4 (80%)	0 (0%)	1 (20%)
Hind	2 (100%)	0 (0%)	0 (0%)
Oblique sesamoidean desmitis			
All	4 (100%)	0 (0%)	0 (0%)
Fore	2 (100%)	0 (0%)	0 (0%)
Hind	2 (100%)	0 (0%)	0 (0%)
Patellar desmitis	2 (100%)	0 (0%)	0 (0%)

*DDF* Deep digital flexor, *SDF* Superficial digital flexor, *AL* Accessory (check) ligament

On 92 of 100 questionnaires, the attending veterinarians reported they were satisfied with both the ELAA and the response to therapy. Seventy-two of these 92 questionnaires reported the horse to have returned to or exceeded their previous level of work, 10/92 reported the horse to have returned to work but not perform to previous standards, and 10/92 reported the horse to have not returned to work as a result of the injury and showed no improvement. Six of 100 questionnaires indicated that the attending veterinarian was not satisfied and reported the horse to have not returned to work as a result of the injury and showed no improvement. A satisfaction response was not documented on 2/100 questionnaires.

Questionnaires indicated that one or more adverse reactions occurred following 14/131 local ELAA injections in 13/100 horses. Most reported adverse events were swelling (11) and/or redness (7) at the administration site. Four horses were reported to have an increase in lameness post- ELAA injection, two of those being non-weight bearing. In three of the four cases, the increased lameness was noted within 24 h of ELAA treatment and resolved within 72 h. In the fourth case, an increase in lameness was not noted until 14 days after injection and resolved within 48 h.

### Comparisons to historical work

The outcomes observed of the horses in the current study were compared to published, peer-reviewed clinical studies that described return to work following treatment with other regenerative therapies (Table 3). For some lesion types (deep digital flexor (DDF) tendonitis of the foot/pastern region, collateral ligament desmitis of the distal interphalangeal joint (CLD-DIPJ), and distal sesamoidean desmitis), previous work using regenerative modalities could not be identified. For these lesion types, historical work using conventional therapies was used for comparison (Table 3). No difference in outcome was observed with any comparison except when CLD-DIPJ outcomes of the current study were compared to those reported by Gutierrez‐Nibeyro et al. [29] (*p* = 0.03). This report [29] described the long-term outcome of 20 horses diagnosed with CLD-DIPJ treated with a variety of modalities with 12 of 20 (60%) horses returning to and maintaining their previous level of exercise for at least three months while eight (40%) were described to have a poor outcome.

**Table 3:** Contingency table analysis for outcome of 100 horses diagnosed with tendionitis/desmitis treated with an equine-origin, liquid amnion allograft to those of previously published works

	Lesion type/ location	Returned to or exceeded previous level of work	Returned to work but could not perform to previous standards	No return to work as a result of the injury and no improvement	*p* value
Current study	All	72	10	18	0.06
Scala et al. 2014 [20]		81	12	7	
Current study	Cannon region	54	22		0.2
Renzi et al. 2013 [16]		13	10		
Current study	SBD	20	10		0.4
Castelijns et al. 2011 [21]		5	6		
Van Loon et al. 2014 [17]		15	7		
Current study	PSD	9	4		0.5
Easter et al. 2014 [25]		215	56		
Current study	SDFT	9	5		0.3
Zuffova et al. 2013 [22]		14	8		
Geburek et al. 2016 [23]		8	2		
Witte et al. 2011 [30]		21	13		
Van Loon et al. 2014 [17]		20	23		
Current study	DDFT—cannon	8	0		0.2
Van Loon et al. 2014 [17]		4	2		
Current study	DDFT—foot/ pastern	12	6		0.2
*Smith et al. 2007 [31]		9	11		
Current study	DIPJ- CD	7	1		0.04
*Gutierrez‐ Nibeyro et al. 2009 [29]		8	12		
Current study	DSD	9	1		0.5
*Sampson et al. 2007 [32]		16	5		
*Schneider et al. 2003 [33]		6	3		

*SBD* Suspensory branch desmitis, *PSD* Proximal suspensory desmitis, *DDFT* Deep digital flexor tendon, *SDFT* Superficial digital flexor tendon, *DIPJ-CD* Distal interphalangeal joint collateral desmitis, *DSD* Distal sesamoidean desmitis

^*^Denotes work describing outcomes of lesions treated with conventional therapies

## Discussion

This multi-center clinical trial represents the first study to evaluate local injection of ELAA for treatment of equine tendonitis and desmitis, and reports that 72 of 100 (72%) treated horses returned to or exceeded their previous level of work after at least six months following treatment. No significant difference was identified comparing these results to a similar report describing the outcome of 99 horses with tendon and ligament injuries treated with local injection of a PRP preparation, although significance of superiority for PRP was approached (*p* = 0.06). In that report [20], 81% of the horses returned to their previous use, 12% had clinical improvement but could not perform at the previous level, and 7% were classified as failures (Table 3). In addition to the regenerative modality used, differences in the type and location of lesions, as well as the breed or discipline of horses could account for the differences in outcome percentages between this report [20] and the current study. Outcomes from another controlled clinical study evaluating local injection of MSCs and PRP for the treatment of tendonitis and desmitis lesions in the cannon region of 23 horses treated with MSCs and PRP [16] were not different from those of the current study (*p* = 0.2).

The most common lesion in the current study was categorized as suspensory branch desmitis (SBD), representing 38 of the 128 (30%) lesions reported. A prior case series described 11 horses with 18 SBD lesions treated with a single intra-lesional injection of PRP reported that five horses (45%) returned to their previous level of work within three months [21]. In another study, 22 warmblood sport horses were treated for SBD using intra-lesional allogeneic umbilical cord-derived MSCs resulting in 15 (68%) had returning to at least the same level of work after six months or more [17]. In the current study, 25 of 38 (66%) SBD lesions were associated with horses that either returned to or exceeded their previous level of work following local injection of ELAA [1]. Outcomes reported in these prior studies [17, 21] compared to those of the current study were not different (*p* = 0.4), thus treatment with ELAA should result in similar outcomes for horses with SBD.

In the current study, proximal suspensory desmitis (PSD) lesions accounted for 15 (12%) of the lesions, and 73% of those were associated with horses that either returned to or exceeded their previous level of work following local injection of ELAA. In a previous study, 107 of 127 (84%) horses with forelimb PSD and 108 of 144 (75%) horses with hindlimb PSD were treated with a series of three peri-ligamentous injections of ACS, entered full training by 110 days from the initial injection, and had no recurrence of lameness after a month of training [25]. In the current study, five of six (83%) forelimb PSD lesions and six of nine (67%) hindlimb PSD lesions were associated with horses that either returned to or exceeded their previous level of work. Although the current study evaluated a smaller population, differences in outcome between this previous study [25] and the current study were not identified (*p* = 0.5), thus treatment with ELAA should result in similar outcomes for horses with PSD.

Superficial digital flexor (SDF) tendonitis accounted for 15 (12%) of the lesions in the current study. A case series of 22 racing Thoroughbreds with SDF tendonitis of varying severity reported that 64% of horses returned to racing following intra-lesional injection with PRP [22]. In a controlled clinical trial of 20 horses with forelimb SDF tendonitis, 8 of 10 (80%) horses treated with intra-lesional injection of PRP were performing at their previous level or higher 12 months post-injury while only 6 of 10 (60%) control horses shared that success [23]. A case series of 40 racing Thoroughbreds with SDF tendonitis treated with a series of four or five intra-lesional injections of insulin-like growth factor-type 1 reported that 21 of 34 (62%) horses for which race data were available raced at least once after treatment [30]. Additionally, 23 warmblood sport horses treated with intra-lesional injection of allogeneic umbilical cord-derived MSCs for SDF tendonitis resulted in 20 (87%) of these horses returning to at least the same level of work at least six months following treatment [17]. In the current study, 67% of the SDF tendonitis lesions treated with local injection of ELAA were associated with horses that returned to or exceeded their previous level of work. The range of success rates reported in these previous reports [17, 22, 23, 30] and the current study may be explained by differences in number, breed and discipline of the horses as well as the location (fore vs hind, proximal vs. distal) and severity of the lesions. Nonetheless, differences in outcome between these previous studies [17, 22, 23, 30] and the current study were not identified (*p* = 0.3), thus treatment with ELAA should result in similar outcomes for horses with SDF tendonitis.

In the current study, DDF tendonitis lesions accounted for 30 of the 128 (20%) lesions and were categorized anatomically as either cannon region or foot/pastern region. All 8 DDF tendonitis lesions in the cannon region as well as 15 of 22 (68%) in the foot/pastern region were associated with horses returning to or exceeding their previous level of work. A study treating six warmblood sport horses with intra-lesional injection of allogeneic umbilical cord-derived MSCs for DDF tendonitis reported that four of these horses (67%) returned to their previous level of work six or more months post-treatment [17], a rate not different (*p* = 0.2) from that of the current study. A review of the current literature could find no reports evaluating the use of regenerative modalities in the treatment of DDF tendonitis of the foot/pastern region exclusively. A retrospective case study of 20 horses with 22 DDF tendonitis lesions treated with endoscopic debridement and/or navicular suspensory desmotomy reported that only nine (45%) returned to pre-treatment performance levels after six or more months [31]. Comparing the outcomes of this report [31] to those of the current study (67% success rate) did not identify a difference (*p* = 0.2) treating similar lesions with ELLA. The low number of horses in both reports could account for the inability to detect a significant difference, and further research using ELLA to treat DDF tendonitis in the foot pastern region may be warranted.

The current report includes 10 horses diagnosed with CLD-DIPJ with 9 of these (90%) returning to or exceeding their previous level of work. This outcome data was better (*p* = 0.03) than that reported by Gutierrez‐Nibeyro et al. [29] who utilized a variety of treatment modalities. Interestingly, one horse in that report was treated with intra-lesional injection of a urinary bladder matrix powder, but the outcome of that specific horses was not described [29]. Based on these outcome data, treatment of DIPJ-CD with ELAA may be warranted but blinded, controlled studies are indicated.

The current study also describes treatment of other collateral (5), distal sesamoidean (11), SDF accessory (2), and patellar (2) desmitis lesions with local injection of ELAA. A search of the current literature found very few reports describing long term outcomes of horses diagnosed with these desmopathies with none evaluating the use of regenerative modalities for treatment. A previous study described 27 horses diagnosed with desmitis of the oblique (18), straight (3), or both (6) sesamoidean ligaments (10 forelimb, 17 hindlimb) treated with various non-regenerative modalities in addition to rest and rehabilitation [32]. Of the 21 horses available for follow-up, 16 (76%) were competing at the same or better level of performance [32]. Another study reported nine cases of straight sesamoidean desmitis (5 forelimb, 4 hindlimb) with six (67%) able to return to their intended use following primarily rest and rehabilitation for six months although four were treated surgically [33]. In the current report, six of seven (86%) horses diagnosed with straight sesamoidean desmitis and all four horses diagnosed with oblique sesamoidean desmitis returned to or exceeded their previous level of work following local injection of ELAA, however, these rates were not different (*p* = 0.5) than previously reported using conventional therapies [32, 33]. In a prior report, six of eight (75%) horses suffering from SDF accessory desmitis returned to their previous level of performance within six months following anti-inflammatory injection of the carpal sheath and rest [34]. Both of the horses diagnosed with SDF accessory desmitis in the current study returned to or exceeded their previous level of work following local injection of with ELAA. In a study of nine clinical cases of patellar desmitis, five were treated with rest and rehabilitation, three were treated surgically, and one was euthanized [35]. Of those nine, only one was able to return to its former level of competition [35]. Both cases of patellar desmitis in the current report returned to or exceeded their previous level of work following local injection of with ELAA. It is the authors’ opinion that the results presented in the current study support the use of local injection of ELAA for these desmopathies.

In the current study, the attending veterinarians reported local adverse reactions in 13% of horses following injection of ELAA. Although most of the events were transient swelling and redness, there were four reports of increase in lameness. In all four cases, the increase in lameness was reported to have resolved within 72 h with non-steroidal anti-inflammatory treatment. In a prior report, a local injection site reaction rate of 4.35% was reported for local injection of allogenic MSCs in 164 horses with 230 lesions, which included 208 tendon or ligament lesions [36]. The disparity in local adverse reaction rate between this report [36] and the current study could relate to the difference in study design or the regenerative product used. Additionally, transient local adverse reaction rates as high as 12% are reported following injection of sodium hyaluronate [37].

The attending veterinarian reported satisfaction with the ELAA product in 92 of 100 horses, indicating an overall positive experience. Interestingly, the attending veterinarian reported satisfaction in 10 horses that failed to show improvement following local injection of ELAA. Reasons for this may be that improvement was not expected due to the severity of the lesion or satisfaction referred to the ELAA product’s availability and ease of administration.

Limitations of the current study include the non-blinded design and the lack of a control group. The longest duration of follow-up was described as 6 months or more, thus some horses many have been evaluated at 6 months, while others may have been evaluated several weeks to months later. Having more precise follow-up periods would have allowed for more uniform reporting of outcomes. In addition, a longer duration of follow-up, perhaps 12 months or more, may have provided relevant information. The number of attending veterinarians likely introduced variability in patient selection and exact treatment methodologies (volume of ELAA injected per lesion, ELAA administration technique, rehabilitation program, adjunct therapies, etc.) and outcome measures utilized; however, the design of the current study did not include collection of these potential variables. Additionally, some of the specific lesion categories were low in numbers and may not represent an appropriate sample size, thus making interpretation difficult.

## Conclusions

Results of this clinical trial indicate that local injection of ELAA achieves comparable rates of return to previous level of work as other regenerative modalities such as MSCs, PRP and ACS in horses diagnosed with tendonitis or desmitis. However, blinded, placebo-controlled studies focusing on specific lesion types are indicated to more accurately evaluate the usefulness of local injection of ELAA for the treatment of equine tendonitis or desmitis.

## Supplementary Information

**Additional file 1. **Veterinarian questionnaire used to gather data following injection of a liquid amnion allograft for the treatment of equine tendonitis and desmitis.

## Data Availability

The data that supports the findings of this study are available upon request of the corresponding author. The data is not publicly available due to client/patient privacy restrictions.

## Abbreviations

ACS: Autologous conditioned serum
DIPJ-CD: Distal interphalangeal joint collateral desmitis
DDF: Deep digital flexor
ELAA: Equine-origin, liquid amnion allograft
MSC: Mesenchymal stem cells
PRP: Platelet rich plasma
PSD: Proximal suspensory desmitis
SBD: Suspensory branch desmitis
SDF: Superficial digital flexor
