# Supplementary material for: Outcome following local injection of a liquid amnion allograft for treatment of equine tendonitis or desmitis – 100 cases
Source: BMC Vet Res. 2022 Nov 7;18:391. doi: 10.1186/s12917-022-03480-5 (PMC9639279; doi:10.1186/s12917-022-03480-5)
Supplement: Supplementary file 1 — Additional file 1. Veterinarian questionnaire used to gather data following injection of a liquid amnion allograft for the treatment of equine tendonitis and desmitis. [file 12917_2022_3480_MOESM1_ESM.pdf]

QUESTIONNAIRE

Thank you for completing this questionnaire to allow us to investigate and report outcome of horses with soft tissue injuries using the liquid amnion allograph product RenoVō™.

Your Full Name ____

Facility ____

Date of Product Administration: ____________________________

Product Serial Number ____________________________

Product Code _______________________

Horse Information

1. Horse Identification ________________________
2. Weight:
   - ☐ < 500 kg
   - ☐ 500 – 800 kg
   - ☐ > 800 kg
3. Age:
   - ☐ ≤ 3 years
   - ☐ 4 – 10 years
   - ☐ ≥ 11 years
4. Breed:
   - ☐ AQHA/APHA
   - ☐ Thoroughbred
   - ☐ Other (Please specify __________________)
5. Sex:
   - ☐ Stallion
   - ☐ Gelding
   - ☐ Mare
6. Discipline:
   - ☐ Western (☐ reining ☐ cutting ☐ working cow)
   - ☐ Show
   - ☐ Sport (☐ dressage ☐ eventing ☐ hunter/jumper ☐ racing)
   - ☐ Other (Please specify (__________________)
7. Type of Injury: ____

8. Duration of Injury:
   - ☐ < 6 months
   - ☐ 6 months – 1 year
   - ☐ 1 – 2 years
   - ☐ > 2 years

9. Lameness Grade (AAEP grading scale):
   a. Pre-Treatment ☐ 1 ☐ 2 ☐ 3 ☐ 4 ☐ 5
   b. ____days Post-Treatment ☐ 1 ☐ 2 ☐ 3 ☐ 4 ☐ 5
   c. ____days Post-Treatment ☐ 1 ☐ 2 ☐ 3 ☐ 4 ☐ 5
   d. ____days Post-Treatment ☐ 1 ☐ 2 ☐ 3 ☐ 4 ☐ 5
   e. ____days Post-Treatment ☐ 1 ☐ 2 ☐ 3 ☐ 4 ☐ 5

10. Did the horse experience any of the following reactions upon administration of the product and if so, when did those reactions **resolve**?

Symptoms	1-3 hrs	Within 24 hrs	Within 48 hrs	Other (Please Specify)	Did Not Experience
Non-weight bearing	☐	☐	☐	☐ ____________________________	☐
Swelling	☐	☐	☐	☐ ____________________________	☐
Fever	☐	☐	☐	☐ ____________________________	☐
Redness or Warmth	☐	☐	☐	☐ ____________________________	☐
Other: ___________	☐	☐	☐	☐ ____________________________	☐

11. Work activity level post product administration?
   - ☐ Returned to or exceeded original level of work
     - ☐ < 3 months ☐ 3 – 6 months ☐ > 6 months
   - ☐ Returned to work but could not perform to previous standards or required more maintenance
     - ☐ < 3 months ☐ 3 – 6 months ☐ > 6 months
   - ☐ No return to work as a result of the injury and no improvement

12. Were you satisfied with the product and response to therapy?
   - ☐ Yes
   - ☐ No
